# Association between ADAM metallopeptidase domain 33 gene polymorphism and risk of childhood asthma: a meta-analysis

**DOI:** 10.1590/1414-431X20176148

**Published:** 2017-08-31

**Authors:** F.J. Sun, L.Y. Zou, D.M. Tong, X.Y. Lu, J. Li, C.B. Deng

**Affiliations:** 1 Department of Pediatrics, The Fifth Affiliated Hospital of Guangzhou Medical University, Guangzhou, China; 2 Department of Prevention and Health Care, The Third Affiliated Hospital, Sun Yat-sen University, Guangzhou, China; 3 Department of Traditional Chinese Medicine, The Fifth Affiliated Hospital of Guangzhou Medical University, Guangzhou, China

**Keywords:** Childhood, Asthma, ADAM33, Gene polymorphisms, Asthma risk, Meta-analysis

## Abstract

This study aimed to investigate the association between ADAM metallopeptidase domain 33 (ADAM33) gene polymorphisms and the risk of childhood asthma. The relevant studies about the relationship between ADAM33 gene polymorphisms and childhood asthma were searched from electronic databases and the deadline of retrieval was May 2016. The single nucleotide polymorphisms (SNPs) of ADAM33 (rs511898, rs2280092, rs3918396, rs528557, rs2853209, rs44707, rs2280091 and rs2280089) were analyzed based on several models including the allele, codominant, recessive and dominant models. The results showed that the ADAM33 rs2280091 polymorphism in all four genetic models was associated with an increased risk of childhood asthma. Positive associations were also found between the polymorphisms rs2280090, rs2787094, rs44707 and rs528557 and childhood asthma in some genetic models. This meta-analysis suggested that ADAM33 polymorphisms rs2280091, rs2280090, rs2787094, rs44707 and rs528557 were significantly associated with a high risk of childhood asthma.

## Introduction

Asthma is a common respiratory disorder in both adults and children, characterized by bronchial hyper-responsiveness, airway inflammation, airflow obstruction, wheezing and breathlessness. Nowadays, the prevalence of asthma in children is increasing worldwide and has become one of the major causes of child hospitalization and morbidity (1). This disease can be induced by environmental factors (such as bacterial infections and tobacco smoke) and multiple genetic factors (2–4). Commonly, asthma starts with wheezing, but in young children with dysfunctional maturating immune system, not all wheezing progresses to asthma. It has been reported that environmental factors as well as genetic predisposition play important roles in asthma development in children (5,6). Several candidate genes have been reported to be functionally implicated during the occurrence and development of asthma, such as pro-inflammatory genes, anti-inflammatory genes, airway remodeling genes, immune modulation genes, etc. (7).

The ADAM (a disintegrin and metalloproteinase) family, a subgroup of the metzincin metalloproteinase superfamily, plays an important role in physiologic processes, such as cell migration, cell fusion, fertilization and immune response (8,9). ADAM33 (ADAM Metallopeptidase Domain 33) is an asthma susceptible gene, and is associated with asthma and bronchial hyper-responsiveness (10). It is located on the human chromosome 20p13 and is highly polymorphic, containing over 70 single-nucleotide polymorphisms (SNPs) (11). ADAM33 is typically expressed in bronchial smooth muscle cells and human lung fibroblasts. Alterations in ADAM33 activity may influence the function of these cells, thereby resulting in airway remodeling (12). Moreover, airway obstruction and bronchial hyper-reactivity induced by the occurrence of airway remodeling are closely related to asthma (13). Recently, several ADAM33 polymorphisms have been shown to be associated with childhood asthma. For example, Shalaby et al. (14) reported that the rs511898 homozygous mutant genotype and the rs44707 heterozygous genotype of ADAM33 were significantly associated with the risk of childhood asthma. A recent cohort study reported a positive relationship of rs2243250 and rs2070874 polymorphisms with childhood asthma (7). There was no consistent opinion to explain the effect of ADAM33 polymorphisms on asthma in children.

In this study, we performed a meta-analysis to examine the association between ADAM33 polymorphism and risk of asthma in children. This study may provide new perspectives in explaining the significance of ADAM33 for predicting the risk of childhood asthma.

## Material and Methods

### Data source

Related studies were searched in PubMed (http://www.ncbi.nlm.nih.gov/pubmed/) and Embase (http://www.embase.com). Key words used for retrieving were “childhood asthma” or “pediatric asthma” or “asthma in children” and “ADAM33”. The language was restricted to English. The deadline of retrieval was May 2016.

### Inclusion and exclusion criteria

The included studies met the following inclusion criteria: 1) reported the relationship between ADAM33 polymorphism and risk of asthma in children; and 2) SNP distributions were available in cases and controls for evaluating odds ratio with its 95% confidence interval (CI). Studies were excluded if they were reviews, reports, comments, letters, etc.

### Data extraction

Two investigators independently extracted the useful information using a standardized form. The following items were extracted: the name of the first author, publication year, geographical location, study year, study type, as well as the gender and age information of the participants, allele frequencies, and number of patients and controls in each SNP (rs511898, rs2280092, rs3918396, rs528557, rs2853209, rs44707, rs2280091 and rs2280089). Divergences were settled by discussion with another investigator.

### Statistical analysis

We first examined if genotype distribution in control participants was in accordance with the Hardy-Weinberg equilibrium (HWE) in each study by Pearson's X^2^ test (15). A meta-analysis was performed with the R statistical package, version 3.12 (https://www.r-project.org/). The association strength between children asthma risk and ADAM33 polymorphisms was estimated by odds ratios (OR) and 95%CI (16). Heterogeneity among studies was detected based on the chi-square Q test and *I* ^2^ test. Heterogeneity was significant when the P value was <0.1 or *I* ^2^ >50%, and the random effect model was used to calculate the pooled effect. Otherwise, the fixed effect model was used (17). Publication bias was evaluated by Egger's method (18).

## Results

### Study selection

**Figure 1.**
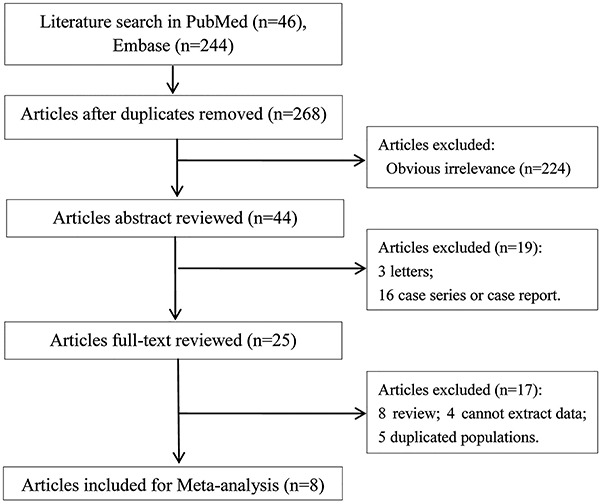
Flow chart of literature search and study selection.

The flow chart of the selection progress is listed in Figure 1. Briefly, 290 articles were preliminarily identified from PubMed (n=46) and Embase (n=244). Of these, 22 duplicate articles were removed. After reading the titles, abstracts and whole test, if possible, another 224 articles were excluded due to obviously irrelevant data. The studies including both adult asthma and children asthma were also excluded. The abstracts of the remaining articles were carefully read, and 19 of them including 3 letters and 16 case series or case reports were excluded. By reading the full text of the remaining 25 articles, 17 were excluded due to duplicated populations or unavailable data. Finally, a total of 8 eligible studies were included in this meta-analysis (7,14, 19–24).

The included studies were published between 2008 and 2016 and were from Saudi Arabia, India, Portugal, Brazil, Czech, Netherlands, Egypt and China (Table 1). There was no significant difference in age and gender among these studies. All were observational studies, including 1 cohort study, 2 cross-sectional pilot studies and 5 case-control studies. The article by Klaassen et al. (7) was based on two types of studies, ADEM (Asthma Detection and Monitoring) study and KOALA study (the Child, Parent and Health: Lifestyle and, Genetic Constitution study). Therefore, the information of these two types of studies were extracted and listed independently in the Tables.

**Table 1.** Characteristics of the included studies.

Author (reference, year)	Study location	Study year	Gender (M/F)		Age (years)		Study design
			Asthma	Control	Asthma	Control	
Al-Khayyat AI (19) 2012	Saudi Arabia	NA	70/37	NA	3-12	3-12	Cross-sectional pilot study
Awasthi S (20) 2011	India	2007 to 2009	143/68	96/41	74.39±45.76 months	73.61±42.56 months	Case-control study
Berenguer AG (21) 2014	Portugal	NA	58/40	NA	13.6±4.3	NA	Case-control study
de Faria ICJ (22) 2008	Brazil	2006 to 2007	NA	NA	NA	NA	Case-control study
Godava M (23) 2012	Czech	2003 to 2005	76/35	NA	0.4-20	NA	Cross-sectional pilot study
Klaassen EM1 (7) 2015	Netherlands	NA	46/30	63/59	6.0±0.1	6.0±0.1	Cohort study
Klaassen EM2 (7) 2015	Netherlands	NA	37/20	108/83	6.5±0.5	6.5±0.6	Cohort study
Qu SQ (24) 2011	China	NA	199/213	192/205	7.74±2.78	7.52±2.95	Case-control study
Shalaby SM (14) 2016	Egypt	NA	215/185	102/98	8.5±3.6	8.8±2.6	Case-control study

Klaassen EM1: data from the study reported by Klaassen based on ADEM (Asthma Detection and Monitoring) study; Klaassen EM2: data from the study reported by Klaassen based on KOALA study (the Child, Parent and Health: Lifestyle and, Genetic Constitution study); NA: not available.

The SNPs of ADAM33 including rs511898, rs2280092, rs3918396, rs528557, rs2853209, rs44707, rs2280091 and rs2280089 were analyzed in this meta-analysis. Distributions of these genotypes in control and in asthmatic children are listed in Table 2. Genotype distributions of almost all of the control populations were consistent with the HWE.

**Table 2.** Distribution of ADAM33 polymorphisms.

Conventional marking (reference)	SNP	Wild type	Asthma				Control				X^2^*	P
			n	WH	HT	MH	n	WH	HT	MH		
Al-Khayyat AI (19)												
T1(T>C)	rs2280091	T	96	38	47	11	86	53	28	5	0.245	0.6204
T2(G>A)	rs2280090	G	94	41	46	7	84	52	25	7	2.144	0.1431
ST+4(A>C)	rs44707	A	99	48	46	5	60	32	28	0	5.558	0.0184
S1(C>T)	rs3918396	C	96	90	6	0	82	80	2	0	0.012	0.9110
Awasthi S (20)												
F+1 (G>A)	rs511898	G	211	39	94	78	137	40	73	24	0.889	0.3458
V4 (C>G)	rs2787094	C	211	34	90	87	137	33	58	46	2.910	0.0880
ST+4(A>C)	rs44707	A	211	38	94	79	137	37	59	41	2.615	0.1058
S2 (C>G)	rs528557	C	211	18	85	108	137	72	51	14	1.156	0.2824
ST+5 (C>T)	Rs597980	C	211	26	94	91	137	33	67	37	0.061	0.8053
Berenguer AG (21)												
V4 (C>G)	rs2787094	C	98	74	23	1	105	80	22	3	0.815	0.3667
S1 (G>A)	rs3918396	G	98	91	7	0	105	95	10	0	0.263	0.6084
de Faria ICJ (22)												
S2 (C>G)	rs528557	C	88	11	38	39	202	11	136	55	37.466	<0.001
Godava M (23)												
F+1 (G>A)	rs511898	G	109	32	58	19	45	15	22	8	<0.001	0.9892
L-1(G>A)	rs2280092	G	109	69	37	3	45	31	12	2	0.324	0.5694
S1 (G>A)	rs3918396	G	109	94	15	0	45	36	8	1	0.384	0.5353
S2 (C>G)	rs528557	C	109	49	46	14	45	21	18	6	0.444	0.5052
S+1 (A>T)	rs2853209	A	109	40	49	20	45	14	22	9	0.004	0.9465
ST+4(T>C)	rs44707	T	109	42	51	16	45	19	19	7	0.060	0.9703
T1 (T>C)	rs2280091	T	109	66	38	5	45	31	12	2	0.324	0.5694
T+1 (C>T)	rs2280089	C	109	68	39	3	45	27	16	2	0.037	0.8467
V-3(G>A)	rs628977	G	109	79	27	3	45	34	9	2	1.370	0.2418
V4(C>G)	rs2787094	C	109	65	41	3	45	27	17	1	0.914	0.3390
V5(A>G)	rs13527	A	109	95	13	1	45	41	4	0	0.097	1.0000
Klaassen EM1 (7)												
F+1 (G>A)	rs511898	G	75	32	38	5	121	40	60	21	0.034	0.8533
S2 (C>G)	rs528557	C	76	41	35	0	122	49	73	0	3.588	0.058
Klaassen EM2 (7)												
F+1 (G>A)	rs511898	G	56	26	21	9	169	76	91	2	23.476	<0.001
S2 (C>G)	rs528557	C	55	35	20	0	176	94	82	0	1.778	0.182
Qu SQ (24)												
F+1 (G>A)	rs511898	G	412	178	198	36	397	173	182	42	0.333	0.5637
T+1 (C>T)	rs2280089	C	412	301	97	14	397	355	39	3	1.980	0.1594
T2(G>A)	rs2280090	G	412	319	86	7	397	326	69	2	0.756	0.3844
T1(T>C)	rs2280091	T	412	140	185	87	397	240	129	28	3.147	0.0761
V4(C>G)	rs2787094	C	412	141	198	73	397	232	134	31	3.259	0.0710
Q-1(G>A)	rs612709	G	412	305	100	7	397	307	87	3	1.620	0.2031
Shalaby SM (14)												
F+1 (G>A)	rs511898	G	400	77	178	145	200	58	107	35	1.427	0.2323
ST+4(A>C)	rs44707	A	400	109	195	96	200	87	84	29	1.362	0.2431

SNP: single nucleotide polymorphism; WH: wild homozygote; HT: heterozygote; MH: mutational homozygote; NOS: Newcastle-Ottawa Scale; n: total number of including subjects. *likelihood-ratio X^2^.

### Meta-analysis

The results regarding the associations between polymorphisms of ADAM33 and asthma risk of children are listed in Table 3 and Supplementary Figures S1-S5. Four genetic models were analyzed for each ADAM33 polymorphism: allele model (wild *vs* mutation), codominant model (heterozygote *vs* wild homozygote, mutational homozygote *vs* wild homozygote), recessive model (wild homozygote *vs* heterozygote+wild homozygote), and dominant model (wild homozygote+heterozygote *vs* wild homozygote).

**Table 3.** Meta-analysis results of association between ADAM33 and childhood asthma.

SNP	K		Test of association OR (95%CI)	Model	Test of heterogeneity^a,b^		
	Cases	Control			*Q*	P	*I^2^* (%)
Allele model							
rs2280089	1044	884	1.68 [0.52–5.42]	Random	11.44	0.0007	91.30
rs2280090	1012	962	1.42 [1.09–1.85]	Fixed	0.19	0.6594	0
rs2280091	1234	1056	2.06 [1.45–2.92]	Random	4.35	0.1134	54.10
rs2787094	1660	1368	1.40 [0.93–2.10]	Random	13.71	0.0033	78.10
rs3918396	606	464	0.82 [0.46–1.47]	Fixed	2.6	0.2722	23.20
rs44707	1638	884	1.48 [1.25–1.75]	Fixed	3.25	0.355	7.60
rs511898	2526	2138	1.22 [0.88–1.68]	Random	29.64	<0.0001	83.10
rs528557	816	728	2.13 [0.70–6.48]	Random	46.01	<0.0001	95.70
Codominant model 1							
rs2280089	153	60	1.36 [0.48–3.88]	Fixed	0.92	0.3379	0
rs2280090	146	103	1.03 [0.43–2.51]	Fixed	2.68	0.1014	62.7
rs2280091	373	204	1.91 [1.24–2.94]	Fixed	1.64	0.4415	0
rs2787094	516	312	1.35 [0.97–1.88]	Fixed	2.11	0.5499	0
rs44707	582	267	1.31 [0.95–1.81]	Fixed	2.08	0.5554	0
rs511898	879	667	1.59 [0.77–3.30]	Random	32.52	<0.001	84.6
rs528557	330	260	2.92 [1.33–6.39]	Random	6.61	0.0366	69.8
Codominant model 2							
rs2280089	386	387	2.01 [0.23–17.8]	Random	3.86	0.0495	74.1
rs2280090	374	387	1.89 [0.78–4.57]	Fixed	1.11	0.2924	9.8
rs2280091	347	359	4.48 [2.93–6.84]	Fixed	3.34	0.1885	40.1
rs2787094	478	453	2.12 [1.07–4.48]	Random	7.29	0.0631	58.9
rs44707	433	252	2.16 [1.52–3.07]	Fixed	3.44	0.3287	12.8
rs511898	676	534	1.71 [0.76–3.85]	Random	34.98	<0.0001	85.7
rs528557	239	159	3.32 [0.30–36.16]	Random	40.06	<0.0001	95
Recessive model							
rs2280089	522	442	1.77 [0.54–5.78]	Random	8.66	0.0033	88.4
rs2280090	506	481	1.50 [1.11–2.02]	Fixed	1.63	0.2021	38.5
rs2280091	617	528	2.65 [2.08–3.38]	Fixed	3.28	0.1944	38.9
rs2787094	830	684	1.56 [0.93–2.64]	Random	12.28	0.0065	75.6
rs3918396	303	232	0.86 [0.47–1.59]	Fixed	2.37	0.3059	15.6
rs44707	819	442	1.68 [1.31–2.16]	Fixed	3.24	0.3556	7.5
rs511898	1263	1069	1.18 [0.88–1.59]	Random	11.51	0.0422	56.5
rs528557	539	662	1.18 [0.34–4.14]	Random	73.05	<0.0001	94.5
Dominant model							
rs2280089	532	442	1.86 [0.25–13.73]	Random	3.29	0.0695	69.6
rs2280090	506	481	1.46 [0.62–3.44]	Fixed	1.92	0.1658	47.9
rs2280091	617	528	3.09 [2.06–4.61]	Fixed	2.44	0.2947	18.2
rs2787094	830	684	1.81 [1.34–2.46]	Fixed	5.68	0.1284	47.2
rs44707	819	442	1.59 [1.17–2.15]	Fixed	2.92	0.4043	0
rs511898	1263	1069	1.62 [0.78–3.37]	Random	36.34	<0.0001	86.2
rs528557	408	364	3.30 [1.09–10.02]	Random	14.97	0.0006	86.6

OR: odds ratio; CI: confidence interval; Codominant model 1: heterozygote *vs* wild homozygote; Codominant model 2: mutational homozygote *vs* wild homozygote. ^a^Random-effects model was used when the P value for heterogeneity test was <0.01, otherwise the fixed-effect model was used. ^b^P<0.10 was considered to be statistically significant for Q statistics.

Heterogeneity test was performed for the selection of a suitable model for pooled effect. The meta-analysis results indicated that all the four models of rs2280091 increased the risk of childhood asthma. In the allele model, the rs2280090, rs2280091 and rs44707 polymorphisms increased the risk of childhood asthma, with OR of 1.42 (1.09-1.85), 2.06 (1.45-2.92) and 1.48 (1.25-1.75) respectively. In the codominant model of heterozygote *vs* wild homozygote, the associations between rs2280091 and rs528557 polymorphisms and asthma in children were significant (OR=1.91, 95%CI=1.24-2.94, and OR=2.92, 95%CI=1.33-6.39, respectively). In the codominant model of mutational homozygote *vs* wild homozygote, significant results were found in 3 polymorphisms: rs2280091 (OR=4.48, 95%CI=2.93-6.84), rs2787094 (OR=2.12, 95%CI=1.01-4.48), and rs44707 (OR=2.16, 95%CI=1.52-3.07). In the recessive model, the rs2280090 (OR=1.50, 95%CI=1.11-2.02), rs2280091 (OR=2.65, 95%CI=2.08-3.38) and rs44707 (OR=1.68, 95%CI=1.31-2.16) also showed an association with high risk of childhood asthma. In the dominant model, four polymorphisms increased the risk of asthma in children: rs2280091 (OR=3.08, 95%CI=2.06-4.61), rs2787094 (OR=1.81, 95%CI=1.34-2.46), rs44707 (OR=1.59, 95%CI=1.17-2.15) and rs528557 (OR=3.30, 95%CI=1.09-10.02).

## Discussion

The present meta-analysis evaluated the relationship between ADAM33 polymorphisms and asthma risk in children. Results showed that in all four genetic models of ADAM33, the rs2280091 polymorphism was associated with the increased risk of childhood asthma. Positive associations were also found between the polymorphisms rs2280090 (allele model and recessive model), rs2787094 (codominant model 2 and dominant model), rs44707 (allele model, codominant model 2, recessive model and dominant model) and rs528557 (codominant model 1 and dominant model) and childhood asthma. These data suggest that these ADAM33 polymorphisms may be causative factors for asthma in children.

ADAM33 was first regarded as a susceptibility gene for bronchial hyper-responsiveness and asthma by a genome-wide linage analysis (25). More than 70 SNPs have been identified in this gene. Some of the asthma-related SNPs are located in regions encoding amino acid changes (26). Others are non-coding SNPs but affect the viability of smooth muscle cells and fibroblasts, affect the inflammation of the airways, and affect the association with other SNPs (26). Therefore, ADAM33 genetic variations may lead to abnormal changes of smooth muscle cells and fibroblasts, thus result in hyper-responsiveness and remodeling of the airway, which is correlated with development of inflammation (13). In a previous meta-analysis, Zheng et al. (27) reported that the ADAM33 rs2280091 polymorphism increased the risk of asthma. The replication of the positive association confirmed the effect of rs2280091 on asthma. However, the meta-analysis by Zheng et al. (27) only illustrated the relationship of one SNP in adults. In the present study, other polymorphisms such as rs2280090, rs2787094, rs44707 and rs528557 were also found to be related to the increased risk of childhood asthma. Although the function of these SNPs in the development of asthma is not fully understood, it is likely that the ADAM33 is an important chemokine in gene mutations that affects the pathogenesis of asthma in children.

Just as other meta-analyses, heterogeneity was found among the articles. The included studies were from different geographical regions, including Asia (Saudi Arabia, India and China), Europe (Portugal, Czech and Netherlands), Africa (Egypt) and America (Brazil), which might contribute to the heterogeneity of genetic diversity. Besides, children in different countries received different medical care, which also influences the phenotype of asthma, and thus might lead to heterogeneity.

Several limitations in this meta-analysis should be pointed out when explaining our results. First, though there might be some confounding factors that affect the results of this meta-analysis, we did not perform subgroup analysis because of insufficient data. Second, only studies selected from databases were included, and thus publication bias might exist. We did not perform the publication bias analysis because eligible studies were less than 10. Third, the control group of some included studies were not ideal since a slight deviation from HWE was found. Therefore, more keywords should be used to retrieve more studies for further evaluate the relationship between ADAM33 polymorphism and childhood asthma.

In conclusion, ADAM33 polymorphisms rs2280091, rs2280090, rs2787094, rs44707 and rs528557 were significantly associated with a high risk of childhood asthma.

## Supplementary material

Click here to view [pdf].

## References

[1] Maziak W, Behrens T, Brasky T, Duhme H, Rzehak P, Weiland S (2003). Are asthma and allergies in children and adolescents increasing? Results from ISAAC phase I and phase III surveys in Münster, Germany. Allergy.

[2] Holgate S, Davies D, Powell R, Howarth P, Haitchi H, Holloway J (2007). Local genetic and environmental factors in asthma disease pathogenesis: chronicity and persistence mechanisms. Eur Respir J.

[3] Chang J-C, Wang L, Chen R-F, Liu C-A (2012). Perinatal gene-gene and gene-environment interactions on IgE production and asthma development. Clin Dev Immunol.

[4] Bottema RW, Kerkhof M, Reijmerink NE, Thijs C, Smit HA, van Schayck CP (2010). Gene-gene interaction in regulatory T-cell function in atopy and asthma development in childhood. J Allergy Clin Immunol.

[5] Chang JC, Wang L, Chen RF, Liu CA (2012). Perinatal gene-gene and gene-environment interactions on IgE production and asthma development. Clin Dev Immunol.

[6] Bottema RWB, Kerkhof M, Reijmerink NE, Thijs C, Smit HA, Schayck CPV (2010). Gene-gene interaction in regulatory T-cell function in atopy and asthma development in childhood. J Allergy Clin Immunol.

[7] Klaassen EM, Penders J, Jöbsis Q, Kd VDK, Thijs C, Mommers M (2015). An ADAM33 polymorphism associates with progression of preschool wheeze into childhood asthma: a prospective case-control study with replication in a birth cohort study. Plos One.

[8] Huovila A-PJ, Turner AJ, Pelto-Huikko M, Kärkkäinen I, Ortiz RM (2005). Shedding light on ADAM metalloproteinases. Trends Biochem Sci.

[9] Yamamoto S, Higuchi Y, Yoshiyama K, Shimizu E, Kataoka M, Hijiya N (1999). ADAM family proteins in the immune system. Immunol Today.

[10] Van Eerdewegh P, Little RD, Dupuis J, Del Mastro RG, Falls K, Simon J (2002). Association of the ADAM33 gene with asthma and bronchial hyperresponsiveness. Nature.

[11] Ito I, Laporte JD, Fiset PO, Asai K, Yamauchi Y, Martin JG (2007). Downregulation of a disintegrin and metalloproteinase 33 by IFN-γ in human airway smooth muscle cells. J Allergy Clin Immunol.

[12] Holgate ST, Davies DE, Powell RM, Holloway JW (2006). ADAM33: a newly identified protease involved in airway remodelling. Pulm Pharmacol Ther.

[13] Lambrecht BN, Hammad H (2012). The airway epithelium in asthma. Nature Med.

[14] Shalaby SM, Abdul-Maksoud RS, Abdelsalam SM, Abdelrahman HM, Almalky MAA (2015). ADAM33 and ADAM12 genetic polymorphisms and their expression in Egyptian children with asthma. Ann Allergy Asthma Immunol.

[15] Schaid DJ, Jacobsen SJ (1999). Biased tests of association: comparisons of allele frequencies when departing from Hardy-Weinberg proportions. Am J Epidemiol.

[16] Liu T, Xu QE, Zhang CH, Zhang P (2013). Occupational exposure to methylene chloride and risk of cancer: a meta-analysis. Cancer Causes Control.

[17] Feng RN, Zhao C, Sun CH, Li Y (2012). Meta-analysis of TNF 308 G/A polymorphism and type 2 diabetes mellitus. Plos One.

[18] Egger M, Minder C (1997). Bias in meta-analysis detected by a simple, graphical test. BMJ.

[19] Al-Khayyat AI, Al-Anazi M, Warsy A, Vazquez-Tello A, Alamri AM, Halwani R (2012). T1 and T2 ADAM33 single nucleotide polymorphisms and the risk of childhood asthma in a Saudi Arabian population: a pilot study. Ann Saudi Med.

[20] Awasthi S, Tripathi P, Ganesh S, Husain N (2011). Association of ADAM33 gene polymorphisms with asthma in Indian children. J Hum Genet.

[21] Berenguer AG, Fernandes AT, Oliveira S, Rodrigues M, Ornelas P, Romeira D (2014). Genetic polymorphisms and asthma: findings from a case-control study in the Madeira island population. Biol Res.

[22] de Faria IC, de Faria EJ, Toro AA, Ribeiro JD, Bertuzzo CS (2008). Association of TGF-beta1, CD14, IL-4, IL-4R and ADAM33 gene polymorphisms with asthma severity in children and adolescents. J Pediatr.

[23] Godava M, Kopriva F, Bohmova J, Vodicka R, Dusek L, Cvanova M (2012). Association of STAT6 and ADAM33 single nucleotide polymorphisms with asthma bronchiale and IgE level and its possible epigenetic background. Biomed Pap Med Fac Univ Palacky Olomouc Czech Repub.

[24] Qu S, Sun D, Wang Y, Zhang C, Lv Y, Yao L (2011). Association of ADAM33 polymorphisms with childhood asthma in a northern Chinese population. Exper Mol Pathol.

[25] Van EP, Little RD, Dupuis J, Del Mastro RG, Falls K, Simon J (2002). Association of the ADAM33 gene with asthma and bronchial hyperresponsiveness. Nature.

[26] Haitchi HM, Powell RM, Shaw TJ, Howarth PH, Wilson SJ, Wilson DI (2005). ADAM33 expression in asthmatic airways and human embryonic lungs. Am J Respir Crit Care Med.

[27] Zheng W, Wang L, Su X, Hu XF (2015). Association between V4 polymorphism in the ADAM33 gene and asthma risk: a meta-analysis. Genet Mol Res.

